# Comparison of Corneal Epithelial Thickness Profiles Between Aqueous-Deficient and Evaporative Dry Eye Disease

**DOI:** 10.3390/jcm15083055

**Published:** 2026-04-16

**Authors:** Yeonwoo Jin, Sangwon Han, Sun Woong Kim

**Affiliations:** Department of Ophthalmology, Yonsei University Wonju College of Medicine, 20 Ilsan-ro, Wonju 26426, Gangwon-Do, Republic of Korea; foxwoo20@naver.com (Y.J.); sw7596@naver.com (S.H.)

**Keywords:** dry eye disease, Sjögren’s syndrome, meibomian gland dysfunction, corneal epithelial thickness, anterior segment optical coherence tomography

## Abstract

**Background/Objectives:** Corneal epithelial thickness (CET) alterations reflect distinct mechanisms in aqueous-deficient and evaporative dry eye disease (DED) subtypes. In this study, we compare the CET profiles between patients with Sjögren’s syndrome (SS) and those with meibomian gland dysfunction (MGD) to elucidate the underlying mechanisms. **Methods:** We retrospectively analyzed 30 patients with SS and 30 age- and sex-matched with MGD. Assessments included corneal staining, Ocular Surface Disease Index (OSDI), tear meniscus height (TMH), non-invasive breakup time, lipid layer thickness (LLT), and anterior segment optical coherence tomography (AS-OCT) CET mapping. Regional CET and superior–inferior asymmetry were compared. **Results:** The SS group exhibited higher corneal staining scores (2.18 ± 1.23 vs. 1.03 ± 1.18, *p* = 0.001) and lower TMHs (0.14 ± 0.06 vs. 0.18 ± 0.07 mm, *p* = 0.013), while the MGD group reported greater OSDI scores (40.39 ± 22.49 vs. 31.25 ± 22.81, *p* = 0.029). A significantly thinner central epithelium (*p* = 0.043) and localized inferior paracentral thinning (2–5 mm zone, *p* = 0.008) were noted in SS. Corneal staining was identified as the primary independent predictor of central and inferior CET reduction in both groups. In the MGD group, LLT was associated with the preserved inferior CET (*p* = 0.045) and superior–inferior thickness difference (*p* = 0.015). **Conclusions:** Distinct structural signatures are observed between DED subtypes. SS features central/inferior thinning from aqueous deficiency-mediated friction, whereas MGD shows a relatively preserved epithelial thickness influenced by LLT. Regional CET analysis may provide mechanistic insights into DED subtyping.

## 1. Introduction

Recognized as a complex disorder affecting the ocular surface, dry eye disease (DED) primarily involves the disruption of tear film homeostasis. Its pathogenesis is driven by interlinked factors, including elevated tear osmolarity, tear film instability, local inflammatory responses, and neurosensory abnormality [1]. Tear film instability results from deficiencies in the aqueous, lipid, or mucin/glycocalyx layers [2].

The main symptoms include dryness, irritation, and discomfort, and the corneal epithelium, as the outermost tissue exposed to hyperosmolarity and mechanical shear from tear film breakup [3,4], directly contributes to such symptoms. The recent literature highlights the growing clinical demand for objective, non-invasive imaging biomarkers to complement traditional subjective symptom scoring in DED management. Advancements in anterior segment optical coherence tomography (AS-OCT) have enabled precise three-dimensional quantification of corneal epithelial thickness (CET) [5]. Affected eyes typically exhibit irregular epithelial topography, characterized by focal thinning [6,7,8] and increased variability [9]. Emerging evidence suggests that DED causes significant spatial alterations in CET, although the reported patterns are often paradoxical depending on the predominant etiology. For example, chronic hyperosmolar and inflammatory stress can induce subclinical edema or compensatory epithelial thickening in certain DED patients [9,10]. Conversely, in states of aqueous deficiency such as Sjögren’s syndrome (SS), pilot investigations have observed pronounced epithelial thinning, which inversely correlates with tear production metrics such as Schirmer’s test [11].

These discrepancies suggest that aqueous-deficient and evaporative DED induce distinct structural damage. While severe volumetric depletion of the tear meniscus in aqueous deficiency leads to direct mechanical friction and accelerated epithelial desquamation, lipid deficiency in evaporative DED is primarily characterized by rapid tear evaporation and the accumulation of inflammatory mediators without immediate total loss of the aqueous cushion.

Despite these known pathophysiological differences, no study has directly compared topographic CET maps between aqueous-deficient (SS) and meibomian gland dysfunction (MGD; evaporative). Therefore, this study aimed to evaluate the spatial CET distribution between SS and MGD to elucidate the subtype-specific structural damage mechanisms.

## 2. Materials and Methods

### 2.1. Study Design and Participants

This study was conducted at the Department of Ophthalmology, Wonju Severance Christian Hospital, Yonsei University. The study protocol was approved by the Institutional Review Board of Wonju Severance Christian Hospital (approval no. CR325126) and adhered to the tenets of the Declaration of Helsinki.

We retrospectively analyzed the medical records of patients diagnosed with DED between January 2022 and December 2024 at our institution. All study participants were assigned to one of the two age- and sex-matched groups.

The SS group included 30 patients (60 eyes) who met the 2016 ACR–EULAR classification criteria for primary SS. This diagnostic framework relies on a cumulative scoring method incorporating seropositivity for anti-SSA/Ro, biopsy-confirmed focal lymphocytic sialadenitis, severe ocular surface damage (indicated by an ocular staining score of ≥5 or Van Bijsterveld score of ≥4), and markedly reduced tear (Schirmer’s test ≤5 mm/5 min) or salivary production (unstimulated flow rate of ≤0.1 mL/min) [12]. The MGD group consisted of 30 patients (60 eyes) diagnosed with MGD based on at least two of the following: an ocular symptom score of ≥3, a lid margin abnormality score of ≥2, or a meibo-score of ≥3, plus quantitative/qualitative abnormalities in meibum secretion [13,14,15].

The exclusion criteria comprised mixed phenotypes (e.g., MGD with a Schirmer’s test of ≤5 mm or combined SS and MGD), a history of ophthalmic surgery, contact lens use within the preceding 6 months, and concurrent non-DED ocular surface inflammatory conditions. Further, to eliminate the confounding effects of topical medications that can alter CET, patients using anti-inflammatory agents (e.g., topical corticosteroids or cyclosporine) or glaucoma medications were excluded. Although some patients used topical lubricants (artificial tears or mucin secretagogues) before referral, these specific agents were confirmed to have no significant macroscopic effect on CET in our preliminary analysis.

### 2.2. Clinical Assessment

All clinical evaluations, including slit-lamp examination, ocular surface staining, and MGD grading, were performed by a single experienced corneal specialist (S.W.K.) at the initial visit to eliminate interobserver variability. The degree of corneal and conjunctival damage was assessed using fluorescein and lissamine green staining and graded using the OSS (Ocular Staining Score) or Van Bijsterveld scoring system. Subjective symptom evaluation was conducted using the Ocular Surface Disease Index (OSDI) questionnaire [1]. Objective tear film parameters were measured using an IDRA ocular surface analyzer (SBM Sistemi, Torino, Italy). The measured parameters included non–invasive break–up time (NIBUT), tear meniscus height (TMH), and lipid layer thickness (LLT).

### 2.3. CET Measurement

CET was measured using anterior-segment optical coherence tomography (AS–OCT; RTVue, Optovue Inc., Fremont, CA, USA). All images were acquired by a single trained and blinded examiner who was unaware of the patients’ clinical diagnoses. To minimize desiccation-induced artifacts and ensure a uniform pre-corneal tear film, patients were instructed to blink naturally immediately before image acquisition. The CET map was acquired within a 9 mm diameter area and analyzed by dividing the cornea into a central region and concentric annular zones. Concentric zones were defined by their distance from the corneal center as follows: Zone 1 (2–5 mm), Zone 2 (5–7 mm), and Zone 3 (7–9 mm). For location–based comparisons, each zone was further subdivided into the superior, inferior, nasal, and temporal sectors.

To evaluate thickness variability on the CET map, the standard deviation (SD) of the CET within the 7 mm zone of each eye was calculated. In addition, the difference between the maximum and minimum CET values (max–min) within the 7 mm zone was calculated.

For zonal analysis, the superior zones (Sup1, Sup2, and Sup3) were defined as the mean epithelial thickness of the superotemporal (ST), superior (S), and superonasal (SN) sectors within each concentric zone. Similarly, the inferior zones (Inf1, Inf2, and Inf3) were defined by the mean thickness of the inferotemporal (IT), inferior (I), and inferonasal (IN) sectors within each zone. The superior–inferior asymmetry in each zone was calculated by subtracting the mean superior epithelial thickness (Sup1, Sup2, and Sup3) from the mean inferior epithelial thickness (Inf1, Inf2, and Inf3), yielding Dif1, Dif2, and Dif3 for Zones 1, 2, and 3, respectively.

### 2.4. Statistical Analysis

All statistical analyses were conducted using IBM SPSS Statistics software (version 28.0; IBM Corp., Armonk, NY, USA). Continuous variables are expressed as means ± SDs. The Kolmogorov–Smirnov test was used to assess data normality.

To appropriately account for within-subject inter-eye correlation, generalized estimating equations (GEEs) with an exchangeable working correlation structure were employed to compare eye-level clinical parameters (e.g., staining score, TMH, and NIBUT) and regional CET profiles between the SS and MGD groups. For patient-level variables, independent t-tests and chi-square tests were used to compare continuous (e.g., age, OSDI score) and categorical (e.g., sex) variables, respectively.

Multivariate GEE regression models were used to isolate independent associations and to control for potential confounders. Specific regional metrics that exhibited significant inter-group differences were designated as dependent variables. Independent variables, including age, corneal staining score, NIBUT, and TMH, were simultaneously incorporated into the models. Statistical significance was set at an alpha level of <0.05. Post hoc power analysis (GEE, α = 0.05) confirmed adequate statistical power for primary outcomes: central CET (effect size = 0.56, power = 97.5%) and inferior paracentral CET (effect size = 0.59, power > 95%), with *n* = 30 patients per group (60 eyes/group).

## 3. Results

### 3.1. Demographics and Clinical Characteristics

The detailed baseline demographics and clinical metrics for both study arms are presented in Table 1. The cohorts were well-matched, with comparable mean ages (57.23 ± 10.53 vs. 57.07 ± 10.61 years in the SS and MGD groups, respectively) and a strong female predominance (96.7% in both groups). Regarding ocular surface integrity, the mean corneal staining score was notably elevated in the SS group compared to the MGD group (2.18 ± 1.23 vs. 1.03 ± 1.18; *p* = 0.001). Conversely, patient-reported discomfort (OSDI) was significantly higher in the MGD group than in the SS group (40.39 ± 22.49 vs. 31.25 ± 22.81; *p* = 0.029). Evaluations of objective tear parameters demonstrated a severely depleted TMH in the SS group (0.14 ± 0.06 mm) compared to the MGD group (0.18 ± 0.07 mm; *p* = 0.013). However, between-group differences in NIBUT (*p* = 0.301) and LLT (*p* = 0.529) were non-significant.

### 3.2. CET Comparison

**Figure 1:**
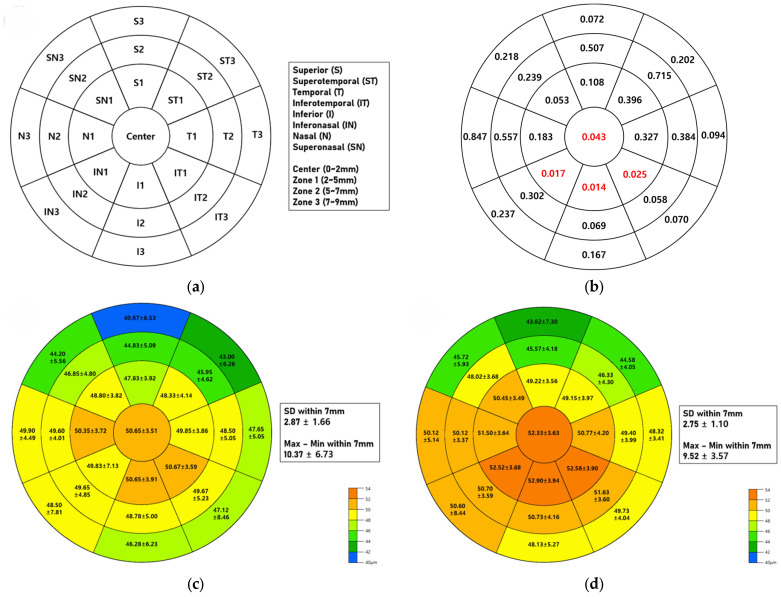
Comparison of topographic corneal epithelial thickness profiles between the Sjögren’s syndrome and meibomian gland dysfunction groups. (**a**) Schematic diagram illustrating the division of the cornea into 25 sectors across a 9 mm diameter map obtained via anterior segment optical coherence tomography. The map consists of a central 2 mm zone (center) and three concentric rings: Zone 1 (2–5 mm), Zone 2 (5–7 mm), and Zone 3 (7–9 mm). Each ring is further subdivided into eight sectors: superior (S), superotemporal (ST), temporal (T), inferotemporal (IT), inferior (I), inferonasal (IN), nasal (N), and superonasal (SN). (**b**) Topographic map displaying the *p*-values from the CET comparison between the SS and MGD groups in each respective sector. Values highlighted in red indicate significant differences (*p* < 0.05). (**c**,**d**) Topographic maps presenting the mean CET (μm) ± standard deviation (SD) for the SS group (**c**) and the MGD group (**d**). The colorimetric scale represents epithelial thickness, ranging from thinner (blue/green) to thicker (yellow/orange) epithelium. Global topographic indices, including the SD and Maximum-Minimum (Max − Min) thickness differences within the 7 mm diameter zone, are displayed in the adjacent boxes.

Figure 1 presents the spatial CET distribution maps, along with the overall thickness variability metrics for both cohorts. After accounting for within-subject inter-eye correlation using the GEE, a topographic comparison revealed a more localized and specific pattern of epithelial thinning in the SS group.

When evaluating individual sectors, the central epithelial layer was significantly thinner in patients with SS than in those with MGD (center, *p* = 0.043). Within the 2–5 mm paracentral ring (Zone 1), significant epithelial thinning was predominantly observed in the inferior sectors of the SS cohort, specifically I1 (*p* = 0.014), IN1 (*p* = 0.017), and IT1 (*p* = 0.025). The superior sectors and outer zones (Zones 2 and 3) did not significantly differ between the two groups.

Furthermore, there were no significant between-group differences in the global topographic indices, including the SD (*p* = 0.673) and Max–Min values (*p* = 0.733) within the 7 mm zone.

Table 2 provides a comprehensive breakdown of the regional CET and vertical asymmetry for both groups. In the zonal analysis, no significant differences in epithelial thickness were noted between the SS and MGD groups across all superior zones (Sup1, Sup2, and Sup3; all *p* > 0.05). In the inferior region, the epithelial thickness was significantly thinner, specifically in the inner zone, in the SS group than in the MGD group (Inf1, 2–5 mm: 50.38 ± 3.97 vs. 52.67 ± 3.75; *p* = 0.008). Although the middle and outer inferior zone epithelia were thinner in the SS group than in the MGD group, the differences were not significant (Inf2, *p* = 0.099; Inf3, *p* = 0.099). Regarding vertical asymmetry, the metrics (Dif1, Dif2, and Dif3) did not significantly differ between the two groups.

### 3.3. Correlations Between CET and Clinical Parameters

Table 3 and Table 4 detail the results of the multivariate GEE regression analyses evaluating the independent clinical factors associated with regional CET in the SS and MGD groups, respectively.

In the SS group, higher corneal staining scores were significantly associated with decreased epithelial thickness in both the central area (Center; B = [−1.317], 95% confidence interval [CI]: −1.923 to −0.710, *p* < 0.001) and the inner inferior zone (Inf1; B = [−1.091], 95% CI: −1.780 to −0.403, *p* = 0.002). Additionally, a higher NIBUT showed a slight positive association with central thickness (*p* = 0.038), whereas older age was associated with decreased superior–inferior asymmetry in the inner zone (Dif1; *p* = 0.023).

Similarly, in the MGD group, higher corneal staining scores were significantly associated with decreased epithelial thickness in the Center (B = [−1.331], 95% CI: −2.198 to −0.465, *p* = 0.003) and Inf1 (B = [−1.155], 95% CI: −2.145 to −0.165, *p* = 0.022) as well as increased structural asymmetry (Dif1: B = [0.501], 95% CI: 0.012 to 0.989, *p* = 0.044). NIBUT was also positively associated with central thickness (*p* = 0.035). Furthermore, a greater LLT was independently associated with increased epithelial thickness in the Inf1 zone (*p* = 0.045) and greater superior–inferior asymmetry (Dif1; *p* = 0.015).

## 4. Discussion

This study used AS-OCT to comparatively analyze CET patterns in patients with SS and MGD. Clinical analysis revealed that the SS group had significantly higher corneal staining scores and lower TMH, whereas the MGD group had higher OSDI scores. Regarding CET mapping, a distinct pattern of thinning localized in the central and inner inferior regions (Inf1) was noted in the SS group compared with the MGD group. Previous studies have reported that the corneal epithelium is typically 2.0–4.0 μm thinner superiorly than inferiorly, with a central thickness of approximately 53–54 μm in healthy eyes [7,16,17,18]. In our study, the SS group exhibited a central CET of 50.65 ± 3.51 μm and an Inf1 thickness of 50.38 ± 3.97 μm, both of which are substantially thinner than these normative values. Conversely, the Inf1 thickness in the MGD group (52.67 ± 3.75 μm) was relatively close to the reported normative range [19,20]. This contrast suggests that the structural impact of SS is markedly more severe and distinct from that of MGD.

Localized inferior and central thinning in the SS group is hypothesized to result from a pathophysiological cascade involving aqueous deficiency and subsequent mechanical friction [21,22,23]. The tear meniscus is essential for maintaining lubrication of the ocular surface and protecting the inferior cornea [24,25]. In healthy individuals, the inferior tear meniscus provides a critical aqueous cushion that buffers friction from the lower eyelid during blinking [19,26]. In this study, the TMH in the SS group (0.14 mm) was significantly lower than that in the MGD group (0.18 mm), indicating a severe depletion of this aqueous cushion. We propose that the collapse of the inferior tear meniscus in SS exposes the inferior cornea to increased mechanical friction, accelerating epithelial desquamation, specifically in the Inf1 zone.

Multivariate GEE regression analysis identified the corneal staining score as the most direct independent predictor of corneal epithelial thinning, as observed in other studies [8,9,27]. Across both groups, higher staining scores were significantly associated with decreased thickness in the Center and Inf1 zones. Notably, although the overall TMH level was significantly reduced in the SS group, it did not emerge as an independent predictor of CET when evaluated simultaneously with the staining score in our multivariable model. This finding strongly aligns with the recognized pathophysiological cascade of DED, wherein aqueous tear deficiency (low TMH) causes a loss of lubrication, leading to increased blink-related friction and subsequent epithelial microtrauma, which manifests clinically as corneal staining [21,28,29]. Our multivariate model suggests a hierarchical relationship in which chronic mechanical desquamation (represented by staining) acts as a proximate mediator that ultimately results in structural thinning, outstripping the basal epithelial proliferation rate [6,28,29]. In SS, the unique vulnerability of the inferior cornea to this cascade was evidenced by persistent significant thinning of the Inf1 zone, even after adjusting for confounders.

In contrast to the SS group, the MGD group exhibited a relatively preserved CET in the inferior region. Notably, our multivariate analysis revealed that greater LLT (consistent with milder MGD severity) was independently associated with increased Inf1 thickness (*p* = 0.045) and superior–inferior difference (*p* = 0.015). This suggests that the preserved lipid layer function mitigates evaporative stress and stabilizes inferior epithelial homeostasis in less severe MGD [30]. The superior–inferior epithelial thickness gradient observed in healthy corneas primarily results from mechanical shear during blinking, preferentially thinning the superior epithelium [31,32]. In SS with profound aqueous deficiency, a diminished TMH compromises the protective aqueous cushion, leading to thinning in the central and inferior regions, thereby reducing the vertical thickness difference. Conversely, patients with MGD and a relatively normal TMH showed similar superior–inferior thickness differences to those of healthy corneas.

Despite the more severe structural damage, the SS group reported lower OSDI scores than did the MGD group. This may be attributed to corneal nerve denervation resulting from chronic severe aqueous deficiency in the SS [33,34]. Conversely, in MGD, chronic exposure to evaporative hyperosmolarity and inflammatory mediators may induce peripheral nerve sensitization and neuropathic hypersensitivity, exacerbating patient discomfort despite relatively mild anatomical thinning [33,35,36].

This study had certain limitations. First, the study lacked a healthy control group, which precluded the establishment of absolute normative references within our specific cohort. Second, while we categorized patients into SS and MGD groups to represent the extremes of aqueous-deficient and evaporative DED, respectively, DED mechanisms frequently overlap in real-world clinical settings. Even within these distinct primary diagnoses, patients may exhibit a mixed-type DED pathophysiology, which could have introduced heterogeneity into our structural analysis. Third, while we excluded patients using topical medications known to directly affect CET (e.g., anti-glaucoma drugs, cyclosporine, or corticosteroids), we included patients using artificial tears or mucin secretagogues. Although these agents do not directly affect CET, they may indirectly influence OCT measurements via tear film stabilization. Finally, due to the lack of biomechanical equipment capable of directly measuring in vivo mechanical friction, friction-related damage was indirectly inferred from the correlations among TMH, staining scores, and epithelial thickness asymmetry.

Despite these limitations, the clinical implications of our findings are significant. Our results suggest that AS-OCT-based spatial CET mapping can serve as a valuable supplementary structural biomarker for DED in routine clinical practice. In severe aqueous-deficient DED such as SS, distinct thinning of the central and inner inferior epithelia can be used as a reliable clinical indicator. Importantly, this regional CET alteration should be interpreted comprehensively, along with other conventional parameters such as TMH and corneal staining scores, to fully capture the underlying pathogenesis.

Conversely, in evaporation-driven conditions, such as MGD, the overall CET may not differ significantly from that of healthy eyes. However, our regional analysis highlights that LLT locally influences the inferior epithelial thickness and vertical asymmetry. This implies that evaluating the regional CET in conjunction with tear film stability parameters can provide a deeper understanding of inferior epithelial homeostasis in evaporative DED.

## 5. Conclusions

In conclusion, AS-OCT-based spatial CET mapping revealed fundamentally distinct structural consequences between aqueous-deficient and evaporative DED subtypes. SS is characterized by pronounced central and inner-inferior epithelial thinning driven by a pathophysiological cascade in which severe aqueous depletion leads to unbuffered blink-related friction and subsequent chronic epithelial microtrauma. Conversely, MGD exhibits a relatively preserved epithelial thickness, with the lipid layer acting as a crucial biomechanical buffer to maintain inferior corneal homeostasis against evaporative stress. Ultimately, the integration of regional CET mapping with conventional clinical parameters may serve as valuable supplementary structural biomarkers. This approach provides objective insights into the predominant biomechanical stressors affecting the ocular surface, thereby facilitating a deeper understanding of the pathophysiology of DED and guiding more targeted management strategies.

## Figures and Tables

**Table 1:** Demographics and clinical characteristics of the Sjögren’s syndrome and meibomian gland dysfunction groups.

Characteristic	SS Group (n = 30, 60 Eyes)	MGD Group (n = 30, 60 Eyes)	*p* Value
Age (years)	57.23 ± 10.53	57.07 ± 10.61	0.931
Female sex, n (%)	29 (96.7%)	29 (96.7%)	1.000
Cornea staining score	2.18 ± 1.23	1.03 ± 1.18	0.001
OSDI score	31.25 ± 22.81	40.39 ± 22.49	0.029
Tear characteristics			
NIBUT	4.75 ± 1.51	5.02 ± 1.00	0.301
TMH	0.14 ± 0.06	0.18 ± 0.07	0.013
LLT	73.95 ± 17.41	71.68 ± 13.73	0.529

Abbreviations: SS, Sjögren’s syndrome; MGD, meibomian gland dysfunction; OSDI, Ocular Surface Disease Index; NIBUT, non-invasive breakup time; TMH, tear meniscus height; LLT, lipid layer thickness.

**Table 2:** Comparison of regional corneal epithelial thickness and superior–inferior asymmetry between the Sjögren’s syndrome and Meibomian gland dysfunction groups.

Characteristic	SS Group (n = 30, 60 Eyes)	MGD Group (n = 30, 60 Eyes)	*p* Value
Superior			
Sup1 (2–5 mm)	48.32 ± 3.86	49.61 ± 3.48	0.133
Sup2 (5–7 mm)	45.88 ± 4.55	46.64 ± 3.78	0.447
Sup3 (7–9 mm)	42.62 ± 5.44	44.64 ± 5.30	0.115
Inferior			
Inf1 (2–5 mm)	50.38 ± 3.97	52.67 ± 3.75	0.008
Inf2 (5–7 mm)	49.37 ± 4.76	51.02 ± 3.61	0.099
Inf3 (7–9 mm)	47.30 ± 6.52	49.49 ± 4.92	0.099
Difference			
Dif1 (2–5 mm)	2.06 ± 3.29	3.06 ± 2.63	0.112
Dif2 (5–7 mm)	3.49 ± 3.23	4.38 ± 3.24	0.218
Dif3 (7–9 mm)	4.68 ± 6.00	4.85 ± 5.40	0.895

**Table 3:** Multivariable regression analysis of factors associated with corneal epithelial thickness in the Sjögren’s syndrome group.

Dependent Variable (Region)	Independent Variables	Unstandardized Coefficient (B)	95% CI for (B)	*p* Value
Center	Age	−0.024	−0.120 to 0.072	0.624
	Corneal Staining Score	−1.317	−1.923 to −0.710	<0.001
	OSDI	0.014	−0.032 to 0.060	0.556
	NIBUT	0.352	0.013 to 0.702	0.038
	TMH	3.562	−11.134 to 18.259	0.635
	LLT	6.769	−0.043 to 0.043	0.998
Inf1	Age	−0.029	−0.154 to 0.096	0.648
	Corneal Staining Score	−1.091	−1.780 to −0.403	0.002
	OSDI	0.018	−0.025 to 0.061	0.402
	NIBUT	−0.086	−0.588 to 0.417	0.739
	TMH	−14.857	−37.992 to 8.279	0.208
	LLT	−0.020	−0.063 to 0.023	0.367
Dif1	Age	−0.110	−0.204 to −0.015	0.023
	Corneal Staining Score	0.244	−0.353 to 0.842	0.423
	OSDI	−0.027	−0.060 to 0.006	0.103
	NIBUT	−0.017	−0.643 to 0.609	0.958
	TMH	18.454	−3.249 to 40.158	0.958
	LLT	0.018	−0.008 to 0.044	0.167

Abbreviations: CI, confidence interval.

**Table 4:** Multivariable regression analysis of factors associated with corneal epithelial thickness in the meibomian gland dysfunction group.

Dependent Variable (Region)	Independent Variables	Unstandardized Coefficient (B)	95% CI for (B)	*p* Value
Center	Age	0.028	−0.068 to 0.123	0.570
	Corneal Staining Score	−1.331	−2.198 to −0.465	0.003
	OSDI	0.019	−0.015 to 0.052	0.275
	NIBUT	0.675	0.014 to 1.337	0.035
	TMH	−4.850	−14.715 to 5.016	0.335
	LLT	−0.011	−0.081 to 0.058	0.754
Inf1	Age	0.029	−0.084 to 0.141	0.616
	Corneal Staining Score	−1.155	−2.145 to −0.165	0.022
	OSDI	0.006	−0.031 to 0.043	0.751
	NIBUT	0.331	−0.417 to 1.079	0.385
	TMH	−1.258	−9.177 to 6.660	0.755
	LLT	0.055	0.001 to 0.109	0.045
Dif1	Age	−0.067	−0.130 to −0.003	0.040
	Corneal Staining Score	0.501	0.012 to 0.989	0.044
	OSDI	0.023	−0.010 to 0.056	0.169
	NIBUT	0.239	−0.417 to 0.895	0.475
	TMH	8.467	−3.255 to 13.679	0.932
	LLT	0.057	0.011 to 0.103	0.015

## Data Availability

The data presented in this study are available on request from the corresponding author. The data are not publicly available due to privacy and ethical restrictions regarding patient medical records.

## Abbreviations

The following abbreviations are used in this manuscript:

AS-OCT	Anterior Segment Optical Coherence Tomography
CET	Corneal Epithelial Thickness
CI	Confidence Interval
DED	Dry Eye Disease
GEE	Generalized Estimating Equation
LLT	Lipid Layer Thickness
MGD	Meibomian Gland Dysfunction
NIBUT	Non-Invasive Breakup Time
OSDI	Ocular Surface Disease Index
OSS	Ocular Surface Staining
SD	Standard Deviation
SS	Sjögren’s Syndrome
TMH	Tear Meniscus Height
